# Cross-scale excitability in networks of quadratic integrate-and-fire neurons

**DOI:** 10.1371/journal.pcbi.1010569

**Published:** 2022-10-03

**Authors:** Daniele Avitabile, Mathieu Desroches, G. Bard Ermentrout

**Affiliations:** 1 Department of Mathematics, Vrije Universiteit Amsterdam, Amsterdam, The Netherlands; 2 MathNeuro Team, Inria at Université Côte d’Azur, Sophia Antipolis, France; 3 Department of Mathematics, University of Pittsburgh, Pittsburgh, Pennsylvania, United States of America; George Mason University, UNITED STATES

## Abstract

From the action potentials of neurons and cardiac cells to the amplification of calcium signals in oocytes, excitability is a hallmark of many biological signalling processes. In recent years, excitability in single cells has been related to multiple-timescale dynamics through *canards*, special solutions which determine the effective thresholds of the all-or-none responses. However, the emergence of excitability in large populations remains an open problem. Here, we show that the mechanism of excitability in large networks and mean-field descriptions of coupled quadratic integrate-and-fire (QIF) cells mirrors that of the individual components. We initially exploit the Ott-Antonsen ansatz to derive low-dimensional dynamics for the coupled network and use it to describe the structure of canards via slow periodic forcing. We demonstrate that the thresholds for onset and offset of population firing can be found in the same way as those of the single cell. We combine theoretical analysis and numerical computations to develop a novel and comprehensive framework for excitability in large populations, applicable not only to models amenable to Ott-Antonsen reduction, but also to networks without a closed-form mean-field limit, in particular sparse networks.

## Author summary

Excitability, the ability of a system to transiently amplify perturbations, is the hallmark of many biological and chemical processes and underlies their ability to send signals. Recent mathematical advances in slow-fast systems provide a rigorous framework for defining excitability in single cells. Here we combine these advances with a recent approach from statistical physics that allows us to extend the notion of excitability to networks of heterogeneous coupled cells. We show through a combination of numerics and analysis that the mechanisms of excitability in single cells pass through to these large networks even when individuals are not themselves excitable.

## Introduction

Excitability is a fundamental *all-or-none* property of many living cells including neurons. For simplicity, we describe neuronal excitability, but this notion extends beyond membrane biophysics. It manifests itself by a very nonlinear response to a sufficiently strong external input, leading to the emission of an action potential before going back to a rest state, whereas any weaker input has no effect on the cell other than a small fluctuation of the membrane potential around its equilibrium value. The concept of excitability is well known to biologists, in particular through the existence of a non-observable boundary in the response space marking the abrupt transition from rest to spike. However, while the geometry of single-cell excitability is well understood [1], the idea of population excitability (for example in a network of coupled neurons) has been far less studied. What makes a population respond normally (such as in working memory tasks [2]) or abnormally (such as in seizures and other pathologies [3]) is a critical question in neuroscience.

Most class-II membrane models [4] —Hodgkin-Huxley, FitzHugh-Nagumo, Morris-Lecar— have a slow-fast structure, with excitability threshold given by so-called *canard solutions* [5–7]. In single cells, canard solutions underpin complex biological rhythms [8], organise transitions from resting to spiking states [9], and from spiking to bursting regimes [10].

The canonical class I excitable systems, such as the QIF neuron models [11], do not intrinsically possess multiple timescales. Nevertheless slow periodic forcing can bring out a bursting rhythm in theta neurons and the threshold to bursting dynamics is again formed by canard solutions [12]. Networks of QIF neurons are capable of generating similar bursting rhythms upon periodic input [13, 14], so the question of network excitability in relation to threshold comes naturally in this context.

In this article, we provide a novel approach to the question of population excitability by showing that the geometry of excitability at the microscopic level scales up to large networks, involving similar key objects related to the slow-fast nature of the system. Excitability in a single neuron is determined by studying how a model responds to small perturbations in the external input or initial conditions: an excitable’s system all-or-none response is mapped in parameter and phase space, by carefully selecting perturbations so that a boundary between markedly different behaviour reveals itself [4, 15, 16]. A major obstacle towards transferring such study from single neuron to population excitability is that it is often unclear how to translate the small perturbations used for single neurons to network and mean field level. In this paper we show that such step is possible and natural for QIF networks, the canonical class I neurons, and that canard solutions are the fabric of excitability thresholds, from single neurons to mean-field descriptions. Dissecting this transition is important, as excitability is a fundamental aspect of the cortex providing a substrate for the propagation of information from one location to another: at the single cell level (via action potentials) and at network level, via propagating waves (for example, see the recent reviews [17, 18] for network models).

Initially, we build on the results by Montbrió et al. [13] (extending previous work by Ott and Antonsen [19]) in the case of dense (all-to-all) networks of QIF neurons, with randomly distributed constant inputs following a heavy-tail (Lorentzian) distribution. For this network, several groups have studied the existence of a simple mean-field limit [13, 20–24]. We show that excitability in large networks of this type is organised via canards in the very same way that it is at the mean-field limit, and we showcase these results computationally, by exhibiting an accurate approximation of the network threshold for networks of size *N* = 10^5^.

What is more, we extend this approach to a much wider class of networks of QIF neurons, including: networks with heterogeneous weights, sparsely-connected networks, networks with electrical as well as chemical synaptic coupling, networks with asymmetrically-spiking neurons, and multi-population networks that combine any of the above features.

The geometry of excitability in all such QIF neural networks beautifully persists across scales, in great generality. For large-enough networks (and up to the mean-field limit) this persistence reveals itself once we consider the correct macroscopic variables, namely the firing rate and the mean membrane potential. This is in contrast to the single-neuron level where the slow-fast variables endowed with this excitable geometry only encompass the membrane potential.

We show that systems of the type above, at any scale, support a continuous route from non-bursting to bursting solutions, upon slow (external) periodic forcing. This continuous route visits canard solutions, which form an interface for excitable transitions, from *down network states* (neural population silent phase) up to *network bursting* —as observed in [13] but without explanation of the threshold transition— as well as for the dual transitions from *up network states* (neural population tonic firing) down to *network bursting* which was not been reported before and involves the same canard geometry and dynamics.

As stated this geometry emerges through a slow periodic forcing. While the best known slow modulatory rhythms in cortex are theta oscillations with frequencies of 5 to 8 Hz, these are not the only slow rhythms that modulate the activity of the cortex. There is a global oscillation that organizes the activity of cortex operating at less than 1Hz and causes a transition from the down state (inactive) to the up state (active). This slow oscillation is present during sleep and anaesthesia (see [25] for a recent review, and also [26, 27]). In addition, there exist slow “delta” oscillations whose frequency ranges from 0.5 − 4 Hz. These have been shown to modulate gamma oscillations in the whisker barrel cortex of awake mice and are coupled to respiration ([28]). These oscillations modulate the excitability allowing gamma and other high frequency oscillations.

In the paper we follow a didactic approach, whereby calculations are performed initially on the original mean field QIF network derived by Montbrió, Pazó, and Roxin in [13], to which we add a synaptic variable as the same group considered in [21]. We henceforth denote this model as the MPR network. With this example we develop intuition and all the technical ingredients to describe the canard population thresholds. We then show how to extend this approach to more general cases.

The paper is organised as follows: in section 1 we introduce the MPR model, present excitability and routes to bursting at single cell and network level; in section 2 we present the mathematical tools to study excitability through folded-saddle canards, and we use them to interpret network excitability, which we showcase numerically in networks of 10^5^ neurons; in section 3, we explain how this approach naturally extends to QIF networks in great generality; in section 4 we demonstrate that the same continuous routes to bursting exist in sparse networks, in the absence of an exact mean field limit for the network; we conclude in section 5.

## 1 Population threshold in MPR networks

### 1.1 QIF Network model

We study a network of *N* all-to-all coupled QIF neurons. The *i*th neuron has membrane potential *V*_*i*_, synaptic variable *s*_*i*_, and is subject to both a background current *η*_*i*_, and an external, zero-mean current *I*(*t*) = *A* sin(*εt*), leading to

$$\begin{array}{c} V_{i}^{\prime }=V_{i}^{2}+\eta_{i}+I\left(t\right)+\frac{J}{N}\sum_{j=1}^{N}s_{j},\;s_{i}^{\prime }=-s_{i}/\tau_{s}, \end{array}$$ (1)

for 1 ⩽ *i* ⩽ *N*. We refer to the sum *K*_*i*_ = *η*_*i*_ + *I*(*t*), as the *(external) input* to the *i*th cell. The ODEs above hold between two consecutive firing times: these are the finite, computable times at which a membrane potential in the network diverges to +∞. Each time this condition is met by *V*_*i*_ we: (i) stop the simulation, (ii) reset *V*_*i*_ to −∞, (iii) send a spike to *all synapses*, whose values are instantaneously incremented by an amount 1/*N*; (iv) restart the simulation of system (1) from these updated initial conditions. A voltage threshold at +∞ is clearly non physical, but one typically addresses this problem in two ways. Firstly, it is possible to use the transformation *V*_*i*_ = tan *θ*_*i*_/2 to obtain

$$\theta_{i}^{\prime }=1-\text{cos}\;\theta_{i}+\left(1+\text{cos}\;\theta_{i}\right)[I\left(t\right)+\eta_{i}+\frac{J}{N}\sum_{j=1}^{N}s_{j}],\;s_{i}^{\prime }=-s_{i}/\tau_{s};$$

one then computes a firing event when *θ*_*i*_ crosses the value *π*/2 from below. Secondly, voltages at *V*_*t*_ = ∞, *V*_*r*_ = −∞ may be replaced by finite, large values *V*_*t*_ = −*V*_*r*_. Below we shall present two types of numerical simulations: for *N* = 1, we use the above transform, hence thresholds are attained at *V* → ∞; for *N* > 1, we use the second strategy instead. The simulation for *N* = 1 in *θ* presents no appreciable difference to the one with *N* = 1 with finite threshold and reset.

This system is ideally suited to study excitability across scales because: (i) we can analyse and compare single cell-dynamics, *N* = 1, network dynamics *N* ≫ 1, and mean-field dynamics *N* → ∞; (ii) one can switch from constant input currents (*ε* = 0) to slowly-varying, oscillatory currents (0 < *ε* ≪ 1) in order to uncover transitions between various cellular regimes.

### 1.2 Single-neuron excitability

**Fig 1:**
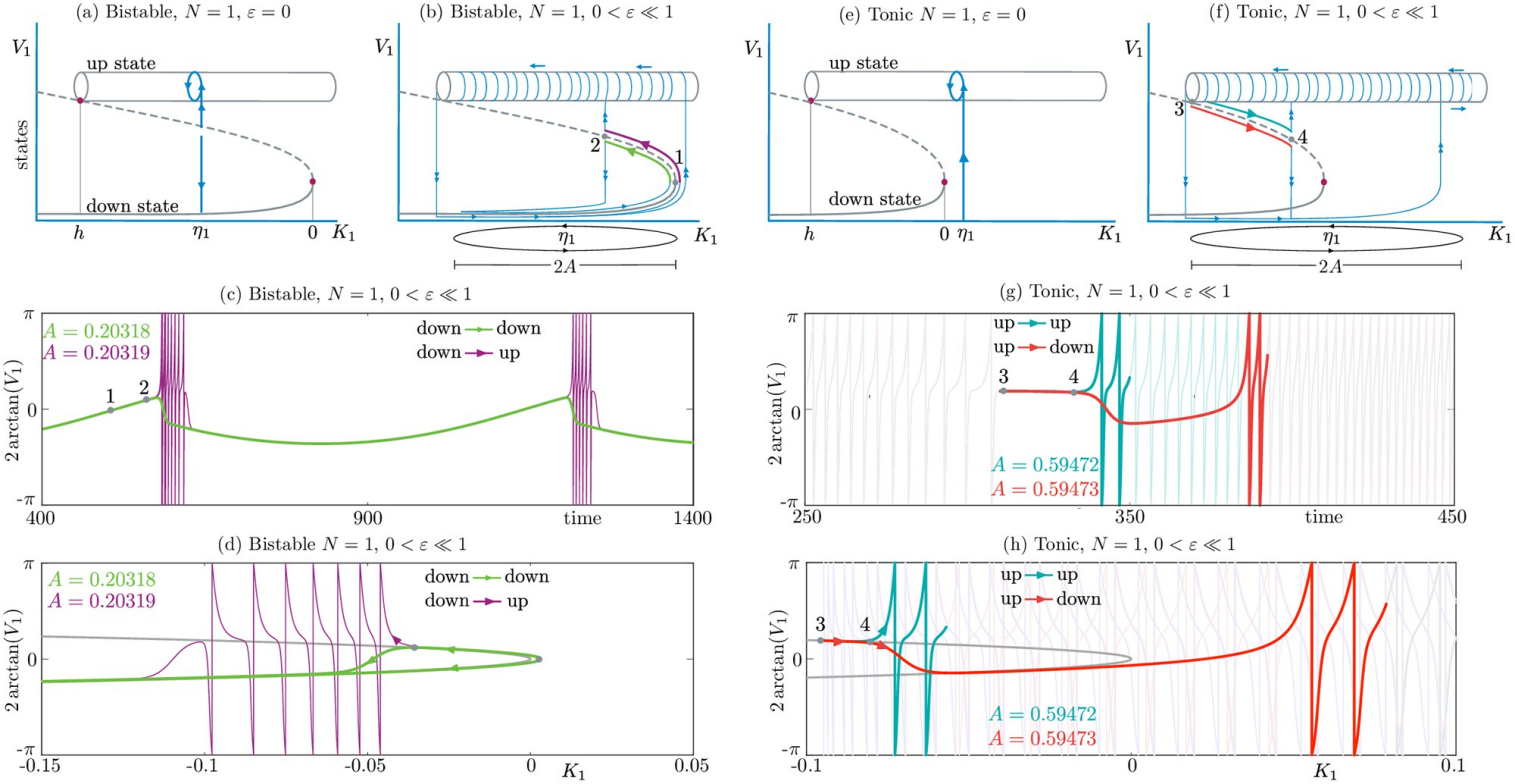
Dynamics of a single QIF neuron (*N* = 1 in Eq (1)) in the bistable regime (a)–(d), and in the tonic regime (e)–(h). (a): Sketch (not to scale) of the bifurcation diagram of steady states (curve) and periodic solutions (cylinder) of the single QIF neuron subject to a constant input *K*_1_ = *η*_1_ (*ε* = 0). A stable quiescent state (down state) coexists with a stable tonic firing solution (up state), separated by an unstable equilibrium (dashed curve). A homoclinic bifurcation is present when *K*_1_ = *h*. In this intrinsically bistable regime (*K*_1_ = *η*_1_ ∈ (*h*, 0)), the cell selects the up or down state depending on initial conditions. (b): When 0 < *ε* ≪ 1, *K*_1_(*t*) = *η*_1_ + *A* sin(*εt*) becomes a slowly varying quantity, oscillating around the value of *η*_1_ (ellipses on the *K*_1_ axes) with amplitude *A*, and transitions between the up and the down phases become possible. The onset between phases is determined by a family of canard solutions 1–2 (see text); in the bistable regime they appear in the down-down (green), and down-up (purple) transitions. (c): Time profiles of two solutions for the system with slow input *K*_1_(*t*), displaying a down-down and down-up transition, containing a canard segment (1–2). (d) The solutions in (c) are plotted in the variables (*V*_1_, *K*_1_), and superimposed on the curve of equilibria of the *ε* = 0 system (grey parabola), providing evidence of canard behaviour (1–2), and part of the orbits greyed out to enhance visibility. Parameters: *ε* = 0.01, *J* = 6, *τ*_*s*_ = 0.3, *η*_1_ = −0.2; *A* values are reported in the panels. (e): Sketch of the bifurcation diagram of steady states and periodic solutions with constant input (*ε* = 0) in the tonic regime *k*_1_ = *η*_1_ > 0). In this regime the cell displays solely the firing solution (up state). (f): When 0 < *ε* ≪ 1 transitions between the up and the down phases become possible, mediated by canard solutions which are possible as up-up and up-down transitions (3–4), but not vice-versa. (g): Time profiles of two solutions in the tonic regime, with slow input *K*_1_(*t*), displaying an up-up and up-down transition, containing a canard segment (3–4). (h): The solutions in (f) are plotted in the variables (*V*_1_, *K*_1_), and superimposed on the curve of equilibria of the *ε* = 0 system (grey parabola), providing evidence of canard behaviour (3–4), and part of the orbits greyed out to enhance visibility. Parameters: *ε* = 0.01 *J* = 6, *τ*_*s*_ = 0.3, *η*_1_ = 0.5; *A* values are reported in the panels.

Let us set *N* = 1, *ε* = 0, and examine a QIF neuron with self-coupled synapse [29], subject to a constant input current *K*_1_ = *η*_1_, as in Fig 1a and 1e. When 0 < *τ*_*s*_ ≪ 1 and *Jτ*_*s*_ is sufficiently large, the cell supports two *coexisting attracting* states: an equilibrium (*down state*), and a periodic solution with tonic firing (*up state*), separated by an intermediate unstable equilibrium. The equilibria belong to a curve which folds when the input *K*_1_ is null; periodic solutions collide with unstable equilibria at a homoclinic bifurcation, when *K*_1_ = *h*. In passing we note that in Fig 1a, 1b, 1e and 1f are only schematics of bifurcation diagrams, without units or scales, which is why they feature “states” on the ordinates. This is in contrast to Fig 1c, 1d, 1g and 1h, which show voltages of computed trajectories.

When *ε* = 0 and *η*_1_ ∈ (*h*, 0) in the intrinsically bistable regime, Fig 1a, initial conditions determine whether the voltage is attracted to the down or to the up state; the threshold is given by the middle unstable state. When *η*_1_ > 0, the only attractor is the periodic solution, hence the cell is intrinsically tonic, Fig 1e. We are interested in how the cell (and later on, the network) transitions from the rest state to the repetitive firing state, and how the two states are concatenated together to form a bursting state. In a standard QIF model without synapses (*J* = 0), there is no bistability (the up state is to the right of the fold): this changes some of the waveforms supported by the cell, but not the mechanisms we aim to describe, namely the bursting transitions between down and up states [12].

To study these transitions, one sets *ε* > 0 small and hence examines slow forcing [1, 4], as sketched in Fig 1b and 1f. This causes the input to oscillate around the mean value *η*_1_ (see ellipses on the horizontal axes). The exact dynamics of the system depends on *A*, the amplitude of the input oscillations, and on the sign of *η*_1_. By varying these two parameters, one can construct a great variety of solutions where up and down states alternate. Some trajectories stand out, in that they signal the onset or termination of a phase. With reference to Fig 1b, small-amplitude forcing with average *η*_1_ ∈ (*h*, 0) causes the cell to oscillate around its rest state; these are subthreshold oscillations, which stick to the down state at all times; upon increasing the amplitude *A* (see ellipse around *η*_1_), the trajectories reach a turning point, when *η*_1_ + *A* ≈ 0, that is, for *A* ≈ −*η*_1_; near this value, there are trajectories which follow the branch of unstable states for increasingly longer times, before jumping to the down state, or to the up state (segments 1–2). A temporal profile of solution jumping down is given in Fig 1c, obtained for *A* = 0.20318, *ε* = 0.01 (green); a profile of a solution jumping up is also in Fig 1c, for *A* to 0.20319 (purple curve). The narrow region of parameter space between *A* = 0.20318 and *A* = 0.20319 contains an entire family of solutions, which spend *O*(1) times near the repelling branch of equilibria of the system with *ε* = 0. This is surprising: when *ε* is small, one would expect the *ε* = 0 analysis to play a role, and therefore trajectories to be repelled exponentially fast from the unstable branch; on the contrary, here we consider trajectories that stay close to the unstable branch for long times. Orbits with such a feature, like the ones labelled with segments 1–2 (and 3–4) in Fig 1 are called *canards*.

These counter-intuitive canard solutions (marked with colors and numbers in Fig 1b), constitute a computable interface between subthreshold oscillations (down-down orbits) and bursting states (down-up orbits). The canards segments in Fig 1b are marked in the time profiles Fig 1c, and are also visible in the phase-plane projection Fig 1d. In the latter the variables (*V*_1_, *K*_1_) are used, and are superimposed on the curve of equilibria of the *ε* = 0 system (grey parabola), providing evidence of canard behaviour (1–2).

The fact that canards occur in such a small range of parameter space makes one wonder whether they are detectable and useful in nature. As we shall see below, canard solutions determine the effective thresholds of the all-or-none responses in a model; they act as excitability thresholds, whose biological relevance is well established [4, 15, 30], even though isolating such threshold may be challenging in experiments. To observe a canard in experiments, one would need access to phase-plane information similar to Fig 1b and 1f, but most electrophysiological experiments record only one observable time trace, like membrane potential, which does not allow for a phase plane interpretation. Nevertheless, time traces can display canard signatures, as we show in Fig 1c and 1g, and similar traits are found in experiments [31], in particular when discussing delayed onset of firing [32]. In addition, analog circuits engineered to reproduce excitability display a clear canard behaviour as they provide access to experimental phase plane data [33].

We then move to the intrinsically tonic regime, for *η*_1_ > 0, Fig 1e–1h. We find up-up and up-down orbits with canards in Fig 1f–1h, whereas it is possible to prove that down-down and down-up canards cannot exist, that is, the transition at the fold is a jump. This is why we have segments of type 3–4, but not of type 1–2, in this scenario. In Fig 1g and 1h show many spikes, most of which are greyed-out for enhancing visibility of the canard segments. In passing we note that segments of type 3–4 are also present in the bistable scenario, but we do not discuss them in the single cell, for the sake of brevity.

The orbits described above capture excitability transitions at a single-cell level. Developing a mathematical understanding of these special orbits is crucial, because canards act as basin boundaries between different cellular responses: biophysical and idealised single-cell models support generically *continuous* canard-mediated transitions [7, 9], as we will exemplify in a moment. The transitions are brutally sharp, but can be continuous, even though they may appear discontinuous upon running simulations. The main contribution of this paper is to show that this scenario also occurs generically and robustly in networks of type-I neurons, of which QIF are universal prototypes.

In addition, the canard-mediated transition from non-bursting to bursting states in networks of QIF neurons transfers across scales: in an isolated QIF cell, as well as in a networks of QIF cells, there exists a continuous route from non-bursting to bursting states, and this path is made of solutions with canard segments such as the ones seen in Fig 1.

**Fig 2:**
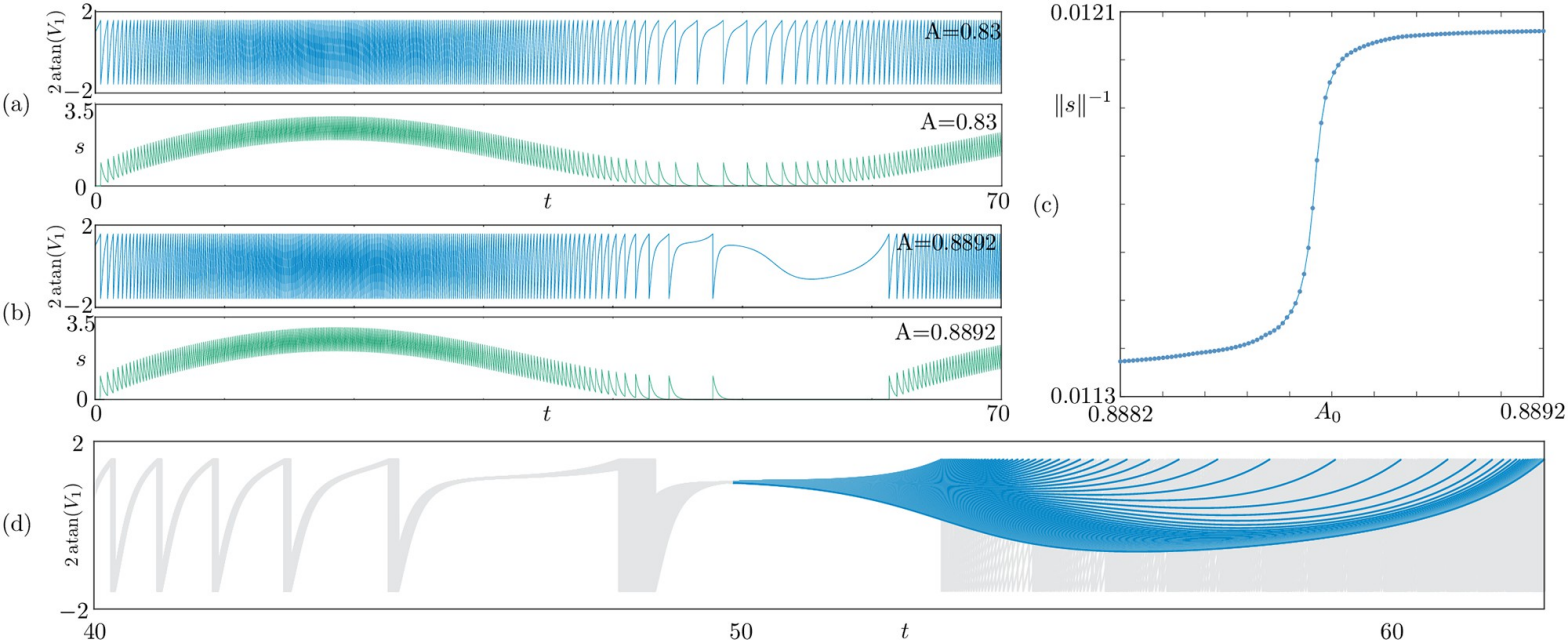
Continuous up-to-down route from non bursting (a) to bursting (b) states of an isolated QIF neuron in the tonic regime, upon increasing the amplitude of *A* of the slow forcing. (a): The non-bursting state is visible when *A* = 0.83; the cell exhibits a tonic state with slow frequency modulations, because the solution hovers on the top branch of Fig 1f, without jumping down. (b): When the forcing amplitude is increased slightly (*A* = 0.8892) we observe a bursting solution, jumping from the top to the bottom branch of Fig 1f. (c): a continuous path connects the orbits in (a,b), as *A* is varied in a narrow band of values; the figure shows the reciprocal of the integral ‖*s*‖ of *s*(*t*) in *t* ∈ [0, 70] as a function of *A*; the sharp increase is typical of canard transitions. (d): we plot 100 solutions along the path in (c), near the sharp increase; the trajectories (grey) are superimposed, and the canard segments are highlighted in blue; with reference to Fig 1h, solutions morph from up-up to up-down states, while growing a canard segment of type 3–4. Parameters: *ε* = 0.1 *J* = 6, *τ*_*s*_ = 0.3, *η*_1_ = 0.5.

Fig 2 shows an example of such transition in a single cell in the tonic regime. When *ε* = 0 the natural solution is a purely tonic one: it locks on the upper branch of Fig 1e. According to Fig 1f, we expect to observe a transition from non-bursting to bursting states, involving solutions with canard segments 3–4, when a slow forcing (*ε* ≠ 0) is switched on. When the forcing has amplitude *A* = 0.83, the cell exhibits a tonic state with slow frequency modulations, as seen in Fig 2a. This solution is a non-bursting state, the frequency modulation is present because the solution hovers on the top branch in Fig 1f, which is composed of periodic orbits with varying period. When the forcing is increased slightly, *A* = 0.8892, we observe a bursting solution, concatenating a tonic spiking phase to a quiescent phase; see Fig 2b.

Fig 2c shows a portion of the continuous path connecting the non-bursting (Fig 2a) to the bursting (Fig 2b) state, as *A* is varied in a narrow band of values. In the plot, we monitor the states using the reciprocal of the integral of *s*, ‖*s*‖ = ∫*s*(*t*)*dt* as *A* varies. The sharp increase along the branch is typical of canard-mediated transitions, and it is called a *canard explosion*.

In Fig 2d we plot 100 solutions along the path, near the canard explosion. The trajectories are superimposed in grey, but we highlight the canard segments in blue: with reference to Fig 1h, solutions morph from up-up to up-down states, while growing a canard segment of type 3–4.

We label the scenario above as a *up-to-down route to bursting*. Other routes to bursting are also possible (from down to up states, for instance) when *η* varies. We do not pursue a classification of bursting routes for a single QIF cell, but we will do so for the mean-field network, after showing that continuous routes to bursting persist across scales, when *N* → ∞.

### 1.3 Network excitability

Let us now consider the network (1), together with reset conditions, subject to random background currents: *η*_*i*_ are taken from the Lorentzian distribution with density $g\left(\eta \right)=\Delta /(\pi {\left(\eta -\bar{\eta }\right)}^{2}+\pi \Delta^{2})$, hence the network is heterogeneous, with some neurons in the bistable regime, and others in the tonic regime. However, the centre of the distribution $\bar{\eta }$ will turn out to play a role. If $\bar{\eta }<0$ ($\bar{\eta }>0$) we say that distribution, or the network, is predominantly bistable (tonic). For *N* → ∞, there is a well-known mean-field limit [13, 21] for the coupled system:

$$\begin{array}{c} \begin{array}{cc} r^{\prime } & =\Delta /\pi +2rv,\\ v^{\prime } & =v^{2}-\pi r^{2}+Js+\bar{\eta }+I\left(t\right),\\ s^{\prime } & =\left(-s+r\right)/\tau_{s}, \end{array} \end{array}$$ (2)

where *r*, *v*, *s* are the mean firing rate, mean membrane potential, and mean synaptic input, respectively. The external input is now given by $K\left(t\right)=\bar{\eta }+I\left(t\right)$. Recall that at microscopic level the background current is constant but heterogeneous (*η*_*i*_, sampled from a Lorentzian distribution), whereas in the mean field limit it is constant and homogeneous (equal to $\bar{\eta }$).

Note that alternative Ott–Antonsen QIF reductions of QIF networks use amplitude and phase of a complex-valued order parameter in place of mean voltage and rate [20, 23]. The order-parameter and rate-voltage descriptions are related through a conformal mapping [13].

**Fig 3:**
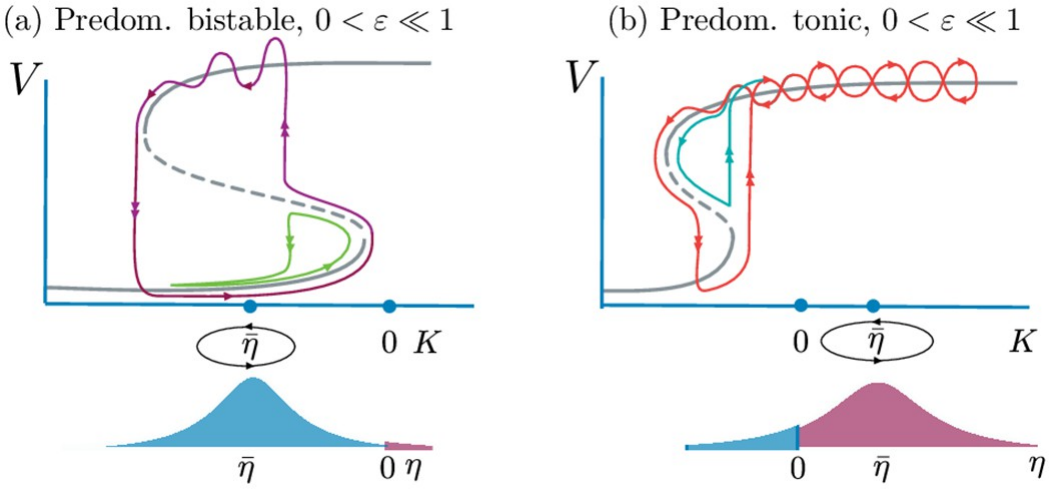
Schematic bifurcation diagram (not to scale, top panels) of the mean-field for the network with randomly-distributed *η*_*i*_ (bottom panels); some neurons are bistable (blue), and others tonic (red), with distribution centred at $\bar{\eta }$. The *ε* = 0 equilibria lie on *S*-shaped curve (grey), whose folds occur for strictly negative values of *K*. The up state is now a high-voltage, high-rate equilibrium. The geometry of excitability persists when *ε* ≪ 1, and *K*(*t*) oscillates slowly around $\bar{\eta }$. (a): Down-down and down-up transitions for predominantly bistable distributions ($\bar{\eta }<0$). (b): Up-up and up-down transitions for predominantly tonic distributions ($\bar{\eta }>0$).

It is important to remark that the mean-field limit introduces a new quantity, the population firing rate, *r* [21] that is not a part of the single or finite system of equations. It emerges in the limit as *N* → ∞ of the microscopic model (1). As seen in Fig 3, when *ε* = 0, hence $K\left(t\right)\equiv \bar{\eta }$, the equilibria of the system lie on an *S*-shaped curve. More precisely, it can be shown that the curve has no folds or 2 folds ([34], Eq 12 and Fig 2(c)). Henceforth we shall assume that *J* is sufficiently large to guarantee the existence of 2 folds, which must occur for negative values of *K*.

In the down state all neurons are close to rest (quiescent network state), whereas the up state corresponds to asynchronous network tonic firing, an averaged version of the tonic state in Fig 1e–1f. The up state can be a stable focus away from the fold and have complex eigenvalues. Between these two stable fixed points is an unstable (saddle) point that serves as a separatrix between the two stable states. Remarkably, when 0 < *ε* ≪ 1, the geometry of excitability still persists in this macroscopic description, and transitions are now determined by the distribution peak at $\bar{\eta }$. We now show that the orbits of the mean field and of the network directly parallel the transitions of the single neuron model, involving the same canard types.

**Fig 4:**
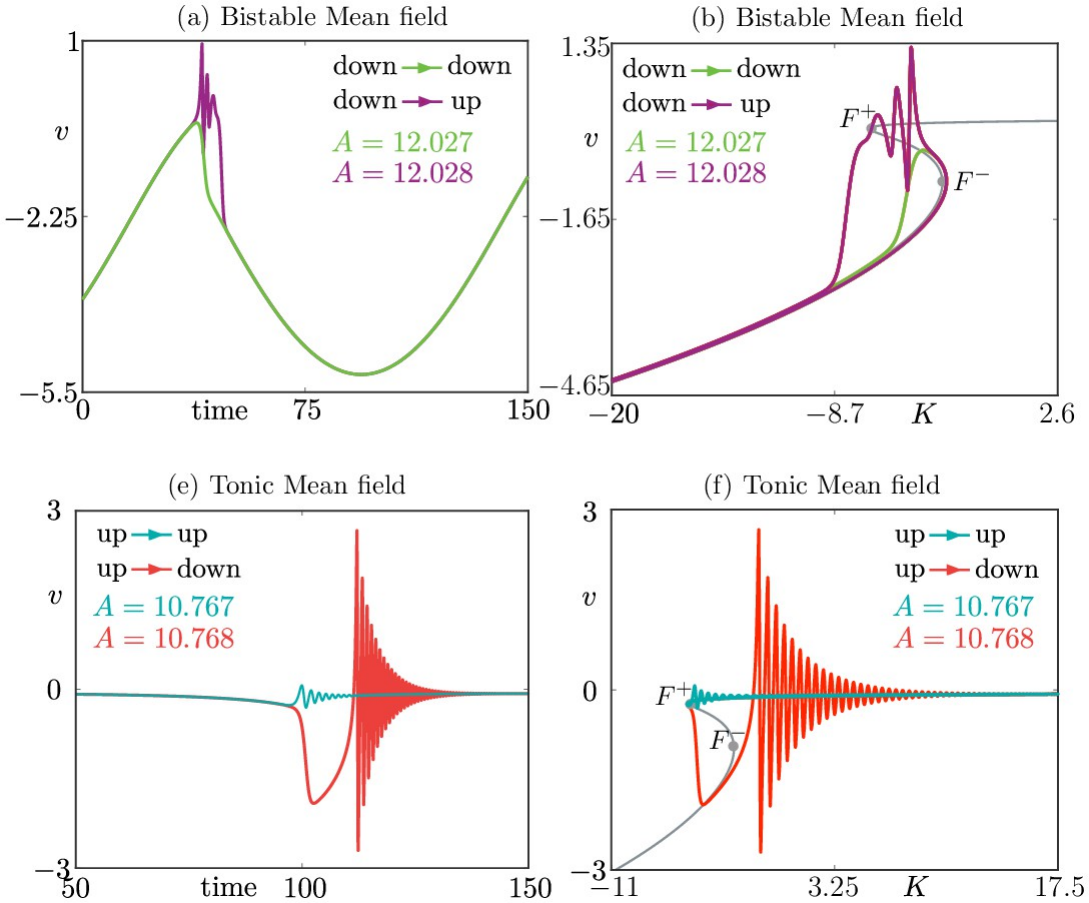
Mean field dynamics of system (2) when the input is slowly varying. Top (bottom) panels show the bistable (tonic) regime, reaching population bursting via two canard scenarios, down-up and up-down, respectively. (a,c): time traces of mean voltage, which display mean-field transitions analogous to the single cell ones in Fig 1e and 1g, respectively. (b,d), same data as in (a,c), plotted in the variables (*v*, *K*), where $K=\bar{\eta }+I\left(t\right)$, superimposed on the folded critical manifold (grey); these figures are the analogues of Fig 1f and 1h, respectively. Parameters are Δ = 1, *J* = 15, *τ* = 0.002, *ε* = 0.05, and (a,b) $\bar{\eta }=-15.1$, (c,d) $\bar{\eta }=5$.

Fig 4b shows a simulation with *ε* = 0.05, $\bar{\eta }=-15.1$ for two different values of the maximum amplitude of the stimulus, *A*, superimposed on the *S*-shaped curve of equilibrium for *ε* = 0 (grey). One trajectory (green) follows the down state, along the bottom branch. The trajectory hugs the unstable branch of fixed points (the canard segment) past the fold *F*^−^, and then jumps down. Small changes in *A* result in a divergence from the down-down (green) to the down-up (purple) states.

The qualitative difference between the two behaviors is more striking in the time traces of the two curves, shown in Fig 4a, where the transition to the up state is accompanied by a burst while for the smaller input, there is just a subthreshold oscillation. The reason for the burst is that the up state is a stable spiral for a range of input values. When $A\gg -\bar{\eta }$, as expected, the network jumps (without canards) from asynchronous to synchronous firing, following the upper branch of the *S*-shaped curve in Figs 3a and 4b.

In conclusion, the trajectories displayed in Fig 4a and 4b are the mean-field equivalents of the single cell ones in the bistable regime, shown in Fig 1c and 1d, respectively. They undergo similar transitions from down-down to down-up states, with canards being the threshold.

The tonic case ($\bar{\eta }=5$) is simulated in Fig 4c and 4d. A similar situation occurs in this case, but the dynamics revolves around the upper fold *F*^+^, and features canards in up-up and up-down orbits.

## 2 Folded-saddle canard behavior across scales

We now derive three central results of the paper: firstly, we characterise the mean-field transitions described above, valid at the ODE level (system (2)), using standard methods from Geometric Singular Perturbation Theory [35]; secondly, we use this characterisation to infer the existence of canard behavior at the network level (system (1)) with *N* = 10^5^ neurons, and provide numerical evidence of this phenomenon; thirdly, we explore canard-mediated routes to bursting at the network level, a feature that persists from the single neuron level. The latter results are remarkable and novel, as canard behavior in large networks is greatly unexplored, in particular for systems with resets and random data.

As anticipated, we initially present the theory for the network described above, and then adapt these results to more general networks. To study the behavior of (2) for small *ε* > 0, we extend the system with two ODEs describing the oscillatory dynamics of *K*(*t*), namely

$$\begin{array}{c} \begin{array}{cc} K^{\prime } & =\;\;\varepsilon Q,\\ Q^{\prime } & =-\varepsilon (K-\bar{\eta }). \end{array} \end{array}$$ (3)

Note that in [13, 24], the mean-field limit model assumes instantaneous synaptic processing, which amounts to taking *τ*_*s*_ = 0 and replacing *s* by *r* in the equations for *r* and *v*. However, assuming 0 < *τ*_*s*_ ≪ 1, that is, a fast synapse, does not change anything about the threshold analysis below while making it more general.

Now that we have expressed the slow dynamics of the current *I* using a second-order harmonic equation, we rescale time in Eqs (2) and (3) so as to parametrise them by the slow time *τ* = *t*/*ε*, as was done in [36], and obtain

$$\begin{array}{c} \begin{array}{cc} \varepsilon \dot{r} & =\Delta /\pi +2rv,\\ \varepsilon \dot{v} & =v^{2}-\pi r^{2}+Js+K,\\ \varepsilon \dot{s} & =\left(-s+r\right)/\tau_{s},\\ \dot{K} & =Q,\\ \dot{Q} & =-(K-\bar{\eta }). \end{array} \end{array}$$ (4)

To shed further light onto the transition from low-rate (down) states to high-rate (up) states of the mean-field, it is key to consider the slow limit of (4) as the forcing speed *ε* tends to 0. Therefore, we set *ε* = 0 in (4) and obtain the three algebraic constraints

$$s=r,\;r=-\frac{\Delta }{2\pi v},\;v^{2}-\pi r^{2}+Js+K=0,$$

In the *ε* → 0 limit, the system variables (*r*, *v*, *s*, *K*, *Q*) evolve on a three-dimensional manifold in $R^{5}$, the so-called *critical manifold*, given by

$$S_{0}=\{s=r=-\frac{\Delta }{2\pi v},\;0=K+\psi \left(v\right)\},$$

where

$$\begin{array}{c} \psi \left(v\right)=v^{2}-\Delta^{2}/\left(4\pi v^{2}\right)-J\Delta /\left(2\pi v\right). \end{array}$$ (5)

**Fig 5:**
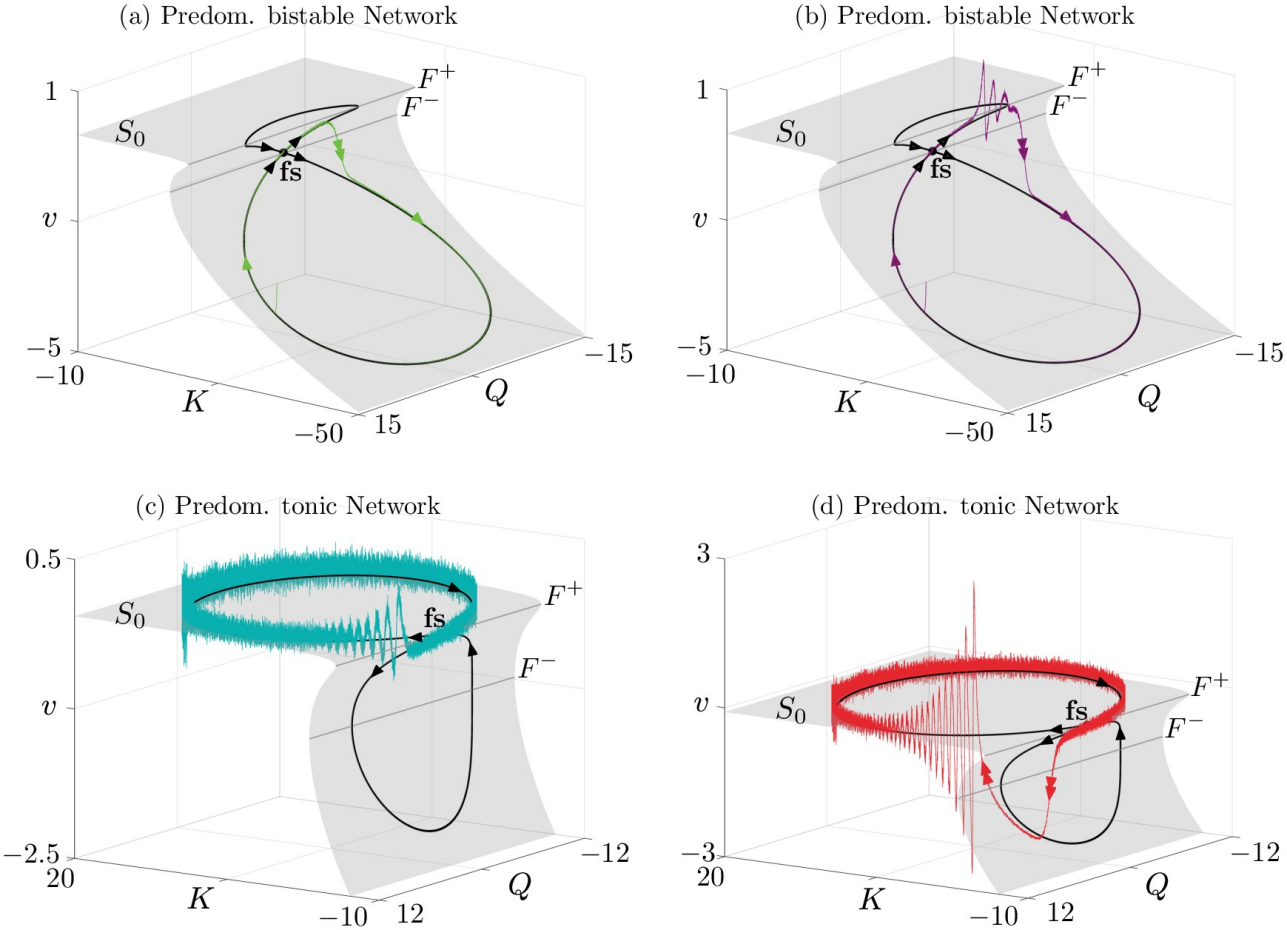
Dynamics of network system (1) with *N* = 10^5^ neurons when the input is slowly varying, in the bistable (a,b) and tonic (c,d) regimes. Shown are the down-down (green) to down-up (purple), and up-up (cyan) to up-down (red) transitions in the network, which mirror the mean-field solutions in Fig 4, as well as the single-cell solutions in Fig 1. To facilitate the comparison between network simulations and mean-field canard theory (see text), network orbits are shown in the 3D phase space (*K*, *Q*, *v*), where *Q* = *I*′. We superimpose them onto the (grey) surface of *ε* = 0 mean-field equilibria, *S*_0_, and fold lines *F*^±^ (also shown in Fig 4b and 4d). On the surface are visible the folded saddle singularity (fs) and its associated canards (black). The networks follows the canard orbits predicted by the mean-field theory remarkably well. Parameters are *Δ* = 1, *J* = 15, *τ* = 0.002, *ε* = 0.05, *V*_*t*_ = 100 = −*V*_*r*_, *A* as in Fig 4, and $\bar{\eta }=-15.1$ (a,b), $\bar{\eta }=5$ (e,f).

The subscript 0 in *S*_0_ refers to the fact that this manifold is found by setting *ε* = 0 in (4). The transitions discussed in this paper occur when *S*_0_ is folded, and it is the union of two attracting and one repelling submanifolds. These conditions occur generically: for common choices of parameters *J*, Δ and $\bar{\eta }$ (see [13], Fig 1(a)), one finds that *S*_0_ has two loci of folds (two lines of folds), *F*^+^ and *F*^−^, corresponding to the set {*Dψ*(*v*) ≔ *ψ*′(*v*) = 0}. A projection of the manifold *S*_0_ onto the (*K*, *v*) plane is visible in Fig 4b and 4d, where the fold lines project onto points *F*^±^; compare with Fig 5 where *S*_0_ is projected onto the (*K*, *Q*, *v*) space and the fold lines are fully visible.

The *ε* → 0 limit introduced above corresponds to a differential-algebraic problem referred to as the *slow subsystem* of the original equation, and in the present case it reduces to

$$\begin{array}{c} \begin{array}{cc} 0 & =K+\psi (v),\\ \dot{K} & =Q,\\ \dot{Q} & =-(K-\bar{\eta }). \end{array} \end{array}$$ (6)

The algebraic constraint in (6) hides the dynamics of *v* in this slow limit. To reveal it, we then differentiate the constraint with respect to time, and obtain the following set of ODEs defined for (*v*, *K*, *Q*) ∈ *S*_0_

$$\begin{array}{c} \begin{array}{cc} -\psi^{\prime }\left(v\right)\dot{v} & =Q,\\ \dot{Q} & =\bar{\eta }+\psi \left(v\right). \end{array} \end{array}$$ (7)

One can relate system (7) to the canards shown in Fig 5: we are considering the *ε* = 0 dynamics, hence we focus on the black curves on *S*_0_, and we take the one in Fig 5a as an example. It would appear that system (7) breaks down at points where *ψ*′(*v*) = 0, that is, along the fold set *F*^±^. Along such folds the first equation reduces to *Q* = 0 hence (7) is undefined at points along the folds where *Q* ≠ 0. However, an inspection of Fig 5a shows that the flow is well defined at one specific point, which is called a folded singularity and is marked as (fs). In fact, there are two other points on *F*^+^ where the trajectory seems to cross the fold. However, these are not associated with canard dynamics, and the flow is not passing through the fold, as the system (7) is singular at those points.

It is around this point that canard solutions are born, because the trajectory passes through (fs) from an attracting to a repelling sheet of *S*_0_. We also note that for *ε* ≪ 1 this behaviour persists (green curve in Fig 5a): slow-fast theory predicts [37] that canards at *ε* = 0 survive for small-enough *ε* > 0 and, as we will see below, they organise the excitable structure of QIF networks.

We therefore investigate the passage through (fs) more precisely: intuitively, at (fs) *ψ*′(*v*) = 0 and *Q* = 0, so that the quotient *Q*/*ψ*′(*v*) stays finite, and the flow of (7) is well defined. We formalise this step by: (i) desingularising (7) with a time rescaling, (ii) identifying (fs) as an equilibrium point of the desingularised problem, (iii) classifying the type of equilibrium, which in turn determines the type of canard in the original system (as done in [36]).

In step (i), we desingularise system (7), and rescale time by a factor −*ψ*′(*v*), which eliminates the prefactor to $\dot{v}$, and regularises the problem, leading to the *desingularised reduced system (DRS)*.

$$\begin{array}{c} \begin{array}{cc} v^{\prime } & =Q,\\ Q^{\prime } & =-\psi^{\prime }\left(v\right)(\bar{\eta }+\psi \left(v\right)). \end{array} \end{array}$$ (8)

An important subtlety is that the time rescaling by −*ψ*′(*v*(*t*)) depends on the state variables *v*, hence the time orientation depends on the position on *S*_0_. In fact, the time rescaling transforming the reduced system (7) into the desingularised reduced system (8) is such that the flow in both systems has the same orientation on the attracting sheets of *S*_0_ but opposite orientation on its repelling sheet.

In step (ii) we look for equilibria of (8), which satisfy *ψ*′(*v*) = 0 and *Q* = 0, hence they geometrically coincide with (fs). Such equilibria are of the form (*v*, *Q*) = (v_*_, 0), where v_*_ satisfies *ψ*′(v_*_) = 0.

In step (iii) we study the linear stability of these equilibria, which is determined by the Jacobian matrix

$$J=[\begin{array}{cc} 0 & 1\\ -\psi^{\prime \prime }\left(v_{*}\right)\psi \left(v_{*}\right) & 0 \end{array}],$$

whose eigenvalues are given by

$$\lambda =\pm \sqrt{-\psi^{\prime \prime }\left(v_{*}\right)\psi \left(v_{*}\right)}.$$

The equilibrium (v_*_, 0) is therefore either a saddle or a center: in the former case the point (fs) is called a *folded saddle*, and gives rise to folded-saddle canards; in the latter case (fs) is a *folded centre*; it is known that this singularity does not give rise to canards, but rather to a discontinuous transition.

A quick calculation reveals that: (1) In the bistable regime, when $\bar{\eta }<0$, there are two (fs) points, one on *F*^+^ and one on *F*^−^, both of which are folded saddles; both folded saddles are visible (in projection) as *F*^+^, *F*^−^ in Fig 4b, whereas only the latter is shown in Fig 5a and 5b. (2) In the tonic regime, when $\bar{\eta }>0$, the singularity on *F*^+^ is still a folded saddle, whereas the one on *F*^−^ is a folded centre, and both are visible in projection in Fig 4d; we show only the folded saddle as (fs) in Fig 5c and 5d. In passing, we note that these findings remain valid for a much larger class of networks and mean field limits, as we will show below.

Fig 5 displays the dynamics of system (7) (*ε* = 0, in black) superimposed on the dynamics of (1) with *N* = 10^5^ (*ε* > 0, in color), for both subthreshold and suprathreshold forcing. Fig 5a shows, in green, the *full network orbit with N* = 10^5^ for *A* slightly below threshold. A canard segment is visible, where the trajectory hugs the black curve on the fold before falling back to the down state. Fig 5b shows the same projection, but for slightly larger *A*; in this case, the trajectory makes the jump to the up state before falling back down. The folded-saddle canards predicted by mean-field theory (in black) are in striking agreement with the behaviour of the network, and the correspondence with the similarly coloured mean-field transitions in Figs 4a, 4b, 1c and 1d is remarkable. These solutions define analogue scenarios to the down-down and down-up states for the single neuron model, but they are exhibited at the level of the network. Interestingly, at single cell level we have a bistable neuron (*η*_1_ < 0), while at the network level some neurons will be tonic, but the network is predominantly bistable ($\bar{\eta }<0$). Similar considerations apply to the predominantly tonic network scenario, showcased in Fig 5c and 5d, and corresponding to Figs 4c, 4d, 1g and 1h.

### 2.1 Continuous routes to bursting

We will now show that for any value of $\bar{\eta }$, the network robustly supports a continuous route from a non-bursting state to a bursting state upon increasing the input amplitude; this transition involves a canard explosion, as seen in Fig 2 for the single cell.

**Fig 6:**
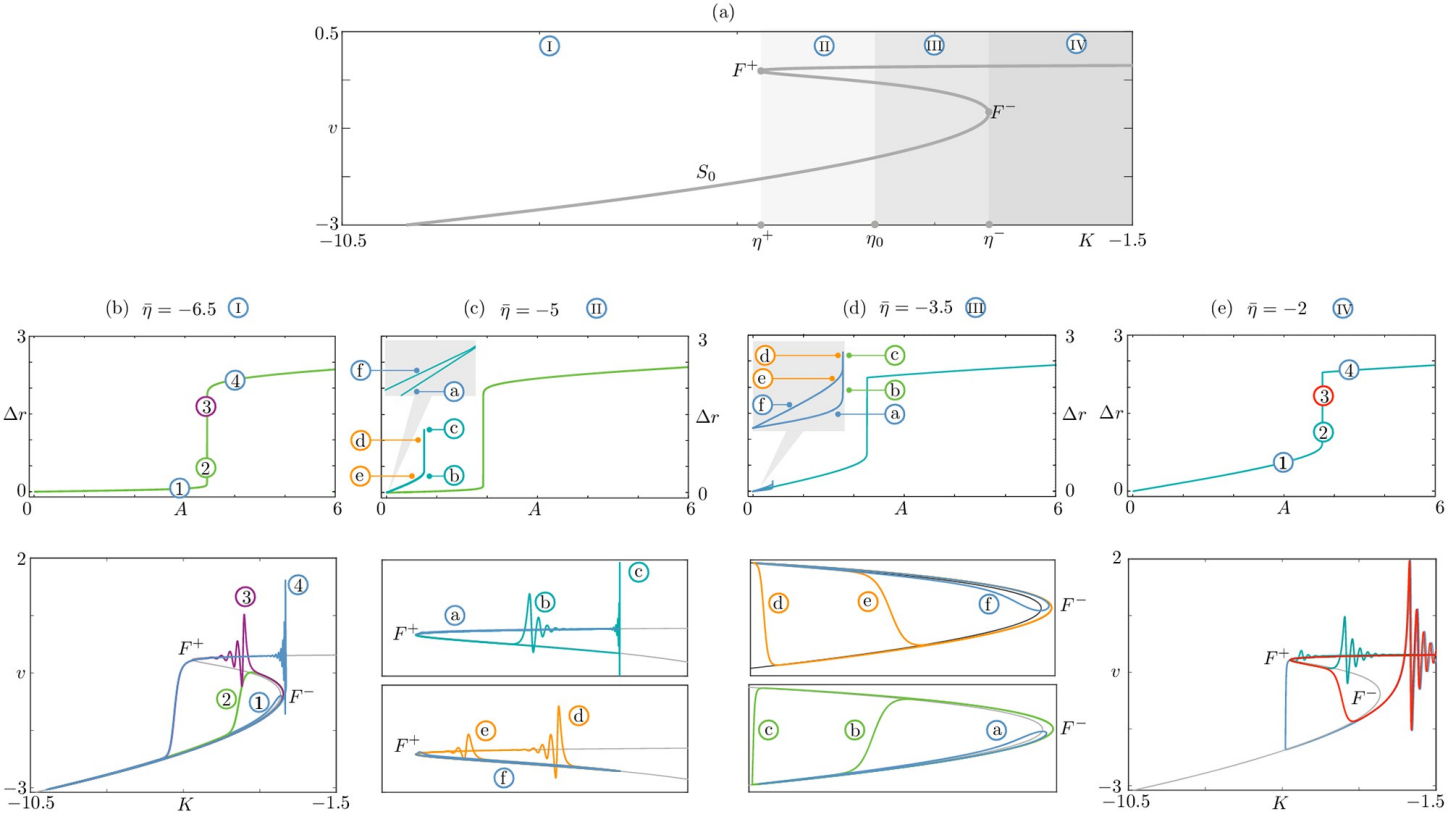
Continuous routes to bursting and non-bursting solution branches, in the mean field model (4) for four different values of $\bar{\eta }$ corresponding to the four different scenarios described in the main text. The associated four regions I, II, III and IV are highlighted on top of the critical manifold *S*_0_ in panel (a). The three values of $\bar{\eta }$ at the transitions between these scenarios are denoted *η*_+_ ≔ *η*(*F*^+^), *η*_−_ ≔ *η*(*F*^−^) and *η*_0_ = (*η*^+^ + *η*^−^)/2, where *F*^±^ are the two folds of *S*_0_. The chosen values of $\bar{\eta }$ in each region are $\bar{\eta }=-6.5$, $\bar{\eta }=-5$, $\bar{\eta }=-3.5$ and $\bar{\eta }=-2$, respectively. Panels (b–d) display both the solution branches obtained by varying the forcing amplitude *A* for a given value of $\bar{\eta }$ (top), and a selection of solutions on the branch, plotted in the phase plane (*K*, *v*) on top of *S*_0_ (bottom). As observed, a continuous branch of solutions bridging from the non-bursting regime to the bursting regime always exists, regardless of the $\bar{\eta }$ value. This branch connects in parameter space a down-down non-bursting solution to a down-up bursting one for $\bar{\eta }<\eta_{0}$ (panels b,c), or an up-up non-bursting solution to an up-down bursting one for $\bar{\eta }>\eta_{0}$ (panels d,e). Additionally, for $\eta^{+}<\bar{\eta }<\eta^{-}$, another solution branch exists, starting at low *A* amplitude and which does not connect to the bursting regime; this branch contains up-up (resp. down-down) solutions for $\eta^{+}<\bar{\eta }<\eta_{0}$ (resp. $\eta_{0}<\bar{\eta }<\eta^{-}$). Parameter values are: Δ = 1, *J* = 15, *τ*_*s*_ = 0.02, *ε* = 0.05; $\bar{\eta }$ and *A* as indicated in the panels.

For sufficiently large coupling values *J*, the critical manifold *S*_0_ is S-shaped, with folds occurring at *η*_+_ < *η*_−_ < 0. As depicted in Fig 6a, there exist 4 different scenarios depending solely on the value of $\bar{\eta }$ with respect to the fold values *η*_±_ and the midpoint *η*_0_ = (*η*_+_+ *η*_−_)/2 between them. This is due to the symmetry of the forcing amplitude around $\bar{\eta }$.

*Case I*: $\bar{\eta }<\eta_{+}$. Since the forcing oscillates around $\bar{\eta }<\eta_{+}$, when a low-amplitude forcing is switched on, the network can only oscillate near the bottom branch of *S*_0_ (orbit 1 in Fig 6b). Upon increasing the amplitude of the forcing, the orbit reaches the fold and hugs the middle unstable branch of *S*_0_ (orbit 2 in Fig 6b). A continuum of orbits with canard segments are now visited by the network: in this transition to bursting visible in the (*A*, Δ*r*) diagram in Fig 6b, the branch of solutions is almost vertical, as *A* varies in a tiny region of parameter space. As we ascend the branch, we pass from a down-to-down solution (green, labelled as 2) to a down-to-up solution (purple, labelled 3) with canard segments. Past the vertical branch, we obtain a fully developed bursting solution (4). The solution branch in Fig 6b reveals a *continuous down-to-up route from non bursting to bursting network solutions*. All solutions with canard segments in this case are of down-to-up or down-to-down type, as witnessed by the green and purple coloring.

*Case II*: $\eta_{+}<\bar{\eta }<\eta_{0}$. Since we enter the bistable region of *S*_0_, we can switch on the forcing near two starting points, on the lower and upper branch, respectively. If one starts from the lower branch, the same considerations as in Case I are valid, and we have a continuous down-to-up route (see green branch in Fig 6c). Starting from the upper branch, low amplitude forcing generate state hovering near the upper branch (solution a in Fig 6c)). When the forcing amplitude increases the orbit grows a canard segment from the upper fold *F*_+_ (solution b). However, due to the proximity between $\bar{\eta }$ and *F*^+^, the branch can only grow canards up to the solution labelled c, and folds back onto itself while displaying solutions d–f. Therefore in Case II there is a continuous down-to-up transition (and no up-to-down) transition to bursting.

*Case III*: $\eta_{0}<\bar{\eta }<\eta_{-}$. This scenario is the mirror image of Case II. The network possesses *continuous down-to-up transition to bursting*, but no down-to-up transition, which is interrupted (Fig 6c). The continuous transition is explained in case IV below.

*Case IV*: $\bar{\eta }>\eta_{-}$. When the forcing is small, the solution can only stay near the upper branch of *S*_0_ (solution 1 in Fig 6e). We can still transition continuously from this non-bursting solution to a bursting solution (solution 4) via canards that start near *F*_+_. This case mirrors Case I, but involves canards of up-to-up and up-to-down type.

## 3 Extension to general QIF networks

### 3.1 Networks with heterogeneous currents

Let us now consider generalisations of the MPR network (1) and mean-field limit (2), for which the excitability scenario described above still holds. We will discuss the generalisations only at the level of the mean field, and refer to existing literature for descriptions of the corresponding microscopic networks.

The starting point is the following generalisation of the MPR mean field (2)

$$\begin{array}{c} \begin{array}{cc} r^{\prime } & =\Delta /\pi +2rv+(\Gamma /\pi -g)s\\ v^{\prime } & =v^{2}-\pi^{2}r^{2}+\left(J+g\;\text{ln}\;a\right)s+\bar{\eta }+I\left(t\right)\\ \tau_{s}s^{\prime } & =(-s+r) \end{array} \end{array}$$ (9)

where: *I*(*t*) = *A* sin(*εt*) is a slow zero-mean periodic forcing as before; Δ, *J*, $\bar{\eta }$ and *τ*_*s*_ are parameters as before; Γ, *g*, and *a* are additional parameters. This generalisation encompasses a variety of exact mean-field limits of QIF networks including:

1. The MPR network with heterogeneous background currents *η*_*i*_ sampled using a Cauchy distribution with peak at $\bar{\eta }$ and half-width at half-maximum (HWHM) Δ. To recover this model from (9) [13, 22] one sets Γ = 0, *g* = 0, *τ*_*s*_ = 0.
2. The MPR network with heterogenous (all-to-all) synaptic coupling [13], obtained for Γ ≠ 0, *g* = 0, *τ*_*s*_ = 0.
3. The MPR network with first-order fast or slow synapses [14, 21] characteristic time *τ*_*s*_, which corresponds to Γ = 0, *g* = 0, *τ*_*s*_ ≠ 0. Note that the analysis done above on (1) assumed 0 < *τ*_*s*_ ≪ 1 (fast synapse), however it is still valid for *τ*_*s*_ = *O*(1) (slow synapse), because the folded-saddle structure only requires the existence of a slow periodic forcing (3). This dynamics also persists with second-order synapses.
4. The modified QIF network from [24] with electrical coupling, which corresponds to Γ = 0, *g* ≠ 0, *a* = 1, *τ*_*s*_ = 0.
5. The modified QIF network studied in [38] with electrical coupling and asymmetric spikes, which differs from the previous case only by *a* ≠ 1.

Before further extending the networks analysable with the proposed formalism, let us rewrite (9) using the generalised coefficients $\tilde{\Gamma }\left(\Gamma ,g\right)=\Gamma /\pi -g$, $\tilde{J}\left(J,g,a\right)=J+g\;\text{ln}\;a$, yielding

$$\begin{array}{c} \begin{array}{cc} r^{\prime } & =\Delta /\pi +2rv+\tilde{\Gamma }s\;≔\;\rho \left(r,v,s\right)\\ v^{\prime } & =v^{2}-\pi^{2}r^{2}+\bar{\eta }+\tilde{J}s+I\left(t\right)\;≔\;\nu \left(r,v\right)+\tilde{J}s+I\left(t\right)\\ s^{\prime } & =\left(-s+r\right)/\tau_{s}\;≔\;\sigma \left(r,s\right). \end{array} \end{array}$$ (10)

It is apparent that one can analyse the slow-fast structure of this system in the exact same way as we have done for system (4), with a cubic-shaped critical manifold given by

$$S_{0}=\{s=r=-\Delta /\left(\pi \left(2v+\tilde{\Gamma }\right)\right),\;0=K+\psi \left(v\right)\},$$

where *ψ* is as in (5) with $\tilde{J}$ instead of *J*. It is apparent that the same folded-saddle dynamics organise the excitable structure of the corresponding mean-field model and can be observed in associated large-enough generalised QIF networks.

Instead of pursuing this analysis, we introduce first a further generalisation, namely we consider the exact mean-field limit of *p* synaptically coupled populations of QIF networks, where we suppose, for simplicity, that only one population (the *k*-th one) receives a slow external periodic forcing. The coupled equations read

$$\begin{array}{c} \begin{array}{cc} \varepsilon {\dot{r}}_{i} & =\rho_{i}\left(r_{i},v_{i},s_{i}\right),\\ \varepsilon {\dot{v}}_{i} & =\nu_{i}\left(r_{i},v_{i}\right)+\sum_{j=1}^{p}{\tilde{J}}_{ij}s_{j}+K\delta_{ik},\\ \varepsilon {\dot{s}}_{i} & =\sigma_{i}\left(s_{i},r_{i}\right),\\ \dot{K} & =Q,\\ \dot{Q} & =-(K-{\bar{\eta }}_{k}), \end{array} \end{array}$$ (11)

for *i* = 1, ⋯, *p*, where *δ* is the Kronecker symbol, and *ρ*_*i*_, *ν*_*i*_, *σ*_*i*_ are the functions defined in system (10) for population-specific choices of parameters ${\tilde{\Gamma }}_{i}$, Δ_*i*_, ${\bar{\eta }}_{i}$, and (*τ*_*s*_)_*i*_. The critical manifold of system (11) is defined by the algebraic constraints

$$\begin{array}{c} \Psi_{i}\left(v_{1},\cdots ,v_{p}\right)\;≔\;\nu_{i}(-\frac{\Delta_{i}/\pi }{2v_{i}+{\tilde{\Gamma }}_{i}},v_{i})-\sum_{j=1}^{p}{\tilde{J}}_{ij}\frac{\Delta_{j}}{\pi (2v_{j}+{\tilde{\Gamma }}_{j})}=0. \end{array}$$ (12)

where

$$\begin{array}{c} r_{i}=s_{i}=-\frac{\Delta_{i}/\pi }{2v_{i}+{\tilde{\Gamma }}_{i}} \end{array}$$

for *i* = 1, …, *p*. Hence, the critical manifold *S*_0_ can be compactly written as

$$\begin{array}{c} S_{0}=\{\left(v_{1},\cdots ,v_{p},K,Q\right)\in R^{p+2}:\\ \;0=\Psi_{i}\left(v_{1},\cdots ,v_{p}\right)+K\delta_{ik},\;i=1,\ldots ,p\}. \end{array}$$ (13)

The expression for *S*_0_ contains *p* independent algebraic conditions in $R^{p+2}$, therefore the critical manifold is indeed a surface, which is consistent with the fact that system (11) has two slow variables. As a consequence, one can write the reduced system associated with (11) in the form

$$\begin{array}{c} \begin{array}{cc} 0 & =\Psi_{i}\left(v_{1},\cdots ,v_{p}\right)+K\delta_{ik},\;i=1,\ldots ,p\\ \dot{K} & =Q\\ \dot{Q} & =-(K-{\bar{\eta }}_{k}) \end{array} \end{array}$$ (14)

The system above mirrors the constrained system (6) in the single-population MPR network. Proceeding like in the single-population case, we differentiate the algebraic constraints with respect to time and project the resulting limiting system onto the (*v*_*k*_, *Q*)-plane to obtain

$$\begin{array}{c} \begin{array}{cc} -\partial_{v_{k}}\Psi_{k}\left(v_{1},\cdots ,v_{p}\right){\dot{v}}_{k} & =Q,\\ \dot{Q} & ={\bar{\eta }}_{k}+\Psi_{k}\left(v_{1},\cdots ,v_{p}\right), \end{array} \end{array}$$ (15)

where *v*_1_, ⋯, *v*_*p*_ satisfy the algebraic constraints defining the critical manifold, that is, the first *p* equations in (14). We recognise in system (15) the same form as (7) for the one-population mean-field limit. We note that the starting system has *p* + 2 equations, but the reduced system (15) has only 2 equations, and it is singular at the fold set of the *k*th population system, given by the condition $\partial_{v_{k}}\Psi_{k}\left(v_{1},\cdots ,v_{k*},\cdots ,v_{p}\right)=0$.

The 2-dimensional system (15) is singular along that fold, and it can be desingularised as in the one-population problem. The folded-saddle and folded-centre classification carries through in this case. Hence we can conclude that the same canard-induced excitability scenario appears in the generic *p*-population case described above.

## 4 Canard transitions across scales in sparse networks

The slow-fast scenarios uncovered in the previous section are valid in a large variety of (all-to-all coupled) QIF networks with exact mean-field limits. We now present evidence that the phenomenon persists in sparse networks, for which no exact mean field limit has been derived to date.

We present this extension for two main reasons: on one hand, we show that the mechanism discussed in the previous section extends further, to sparse networks; on the other, we want to emphasise that the availability of a mean-field description is not strictly necessary for the canard phenomenon, which is supported by generic network systems of QIFs with finite size. In Section (1.3) we studied networks with exact mean fields: since an ODE description was available for the case *N* → ∞, we used these ODEs to predict the region in parameter space where canard dynamics occur, and to classify the folded singularities organising the transition from non bursting to bursting patterns. However, networks with finite size also support canard-mediated transitions, as evidenced in Figs 2 and 4, where the orbits are computed for a very small (*N* = 1) and a very large (*N* = 10^5^) network, respectively.

Having a mean field description at our disposal is useful to pinpoint regions of parameter space where canards will occur, through the study of *S*_0_ and its folded lines; also, large networks of neurons possess canard solutions that are almost indistinguishable from their mean field ones. However, canard mediated transitions are present (and can be documented) in finite-size networks, even when the mean field is inexact, or unavailable in closed form.

To substantiate this claim, we study a sparse network of *N* synaptically-coupled QIF neurons. For a similar network, a heuristic mean field description has been proposed, based on sparsity scaling arguments [22, 39]. The heuristic mean field is in the form (10) and hence canards of folded saddle type are supported by this set of ODEs. However, this mean field is not the exact limit of a network of QIF neurons, and the extent to which the mean field approximates the finite-size network is also immaterial to find canards: as we shall see, both the heuristic mean field and the finite-size network have canard-mediated routes to bursting, even if the two models do not agree well in certain regions of parameter space.

We consider *N* synaptically-coupled QIF neurons of the following form

$$\begin{array}{c} \begin{array}{cc} & V_{i}^{\prime }=V_{i}^{2}+\eta_{i}+I\left(t\right)+\frac{J}{N\sqrt{M}}\sum_{j=1}^{N}W_{ij}s_{j},\\ & \tau_{s}s_{i}^{\prime }=-s_{i}, \end{array} \end{array}$$ (16)

for 1 ⩽ *i* ⩽ *N*, where *M* is an integer controlling the expected number of connections of a neuron. More precisely, the connectivity matrix *W*, with entries *W*_*ij*_, is a binary sparse matrix: the *i*th neuron receives input from *γ*_*i*_ randomly selected neurons; the degree *γ*_*i*_ is also random, given by

$$\gamma_{i}=\lfloor k_{i}\rfloor \chi_{\left[0,2M\right]}\left(k_{i}\right),\;k_{i}\overset{i.i.d}{∼}\frac{\Delta_{\gamma }\sqrt{M}}{{\left(k-M\right)}^{2}+\Delta_{\gamma }^{2}M},$$

where *χ* is the indicator function. In practice, the connectivity matrix is established as follows: a candidate degree *k*_*i*_ is extracted from a Cauchy distribution with center *M* and HWHM $\Delta_{\gamma }\sqrt{M}$ and, if it lies in the interval [0, 2*M*], is rounded to the nearest lower integer to give *γ*_*i*_; the *i*th row of the matrix *W* has *γ*_*i*_ randomly selected entries equal to 1, and the remaining *N* − *γ*_*i*_ entries equal to 0.

In this model, the synaptic input scales as $1/\sqrt{M}$, the external forcing is given by $I\left(t\right)=A\sqrt{M}\;\text{sin}\left(\varepsilon t\right)$, and the background currents *η*_*i*_ are i.i.d, Cauchy distributed with peak at $\bar{\eta }\sqrt{M}$ and HWHM $\Delta \sqrt{M}$. In passing, we note that Δ_*γ*_ ≠ Δ. Finally, the variables *v*_*i*_ and *s*_*i*_ are reset as in the other network examples presented above.

With these scalings for the variable *M* a heuristic, approximate mean-field description was proposed recently [22, 39], for a system of inhibitory neurons with no forcing (*I*(*t*)≡0), homogeneous currents (*η*_*i*_ ≡ *η*) and similar connectivity pattern. Reasoning in a similar fashion, we arrive at the following candidate approximate mean field given by

$$\begin{array}{c} \begin{array}{cc} \varepsilon \dot{r} & =\Delta /\pi +2rv+J\Delta_{\gamma }s/\pi \\ \varepsilon \dot{v} & =v^{2}+\sqrt{M}\left(K+Js\right)-{\left(\pi r\right)}^{2}\\ \varepsilon \dot{s} & =\left(-s+r\right)/\tau_{s}\\ \dot{K} & =Q\\ \dot{Q} & =-(K-\bar{\eta }). \end{array} \end{array}$$ (17)

Differently from [22, 39], the model above has heterogeneous currents, in addition to sparse, heterogeneous connectivity. Also we consider an excitatory neuronal population, as opposed to a inhibitory one. The inhibitory population considered in [22, 39], with *J* = 1 and a term −*Js* in the *v*-equation, is not suitable for studying excitability and transition to bursting, as the critical manifold is not folded. In passing we note that inhibition prevents canard behaviour in this particular model, but not in others: the generalisations of the MPR network given in 3 is valid for coupled populations of excitatory and inhibitory networks, which generically support canard behaviour.

**Fig 7:**
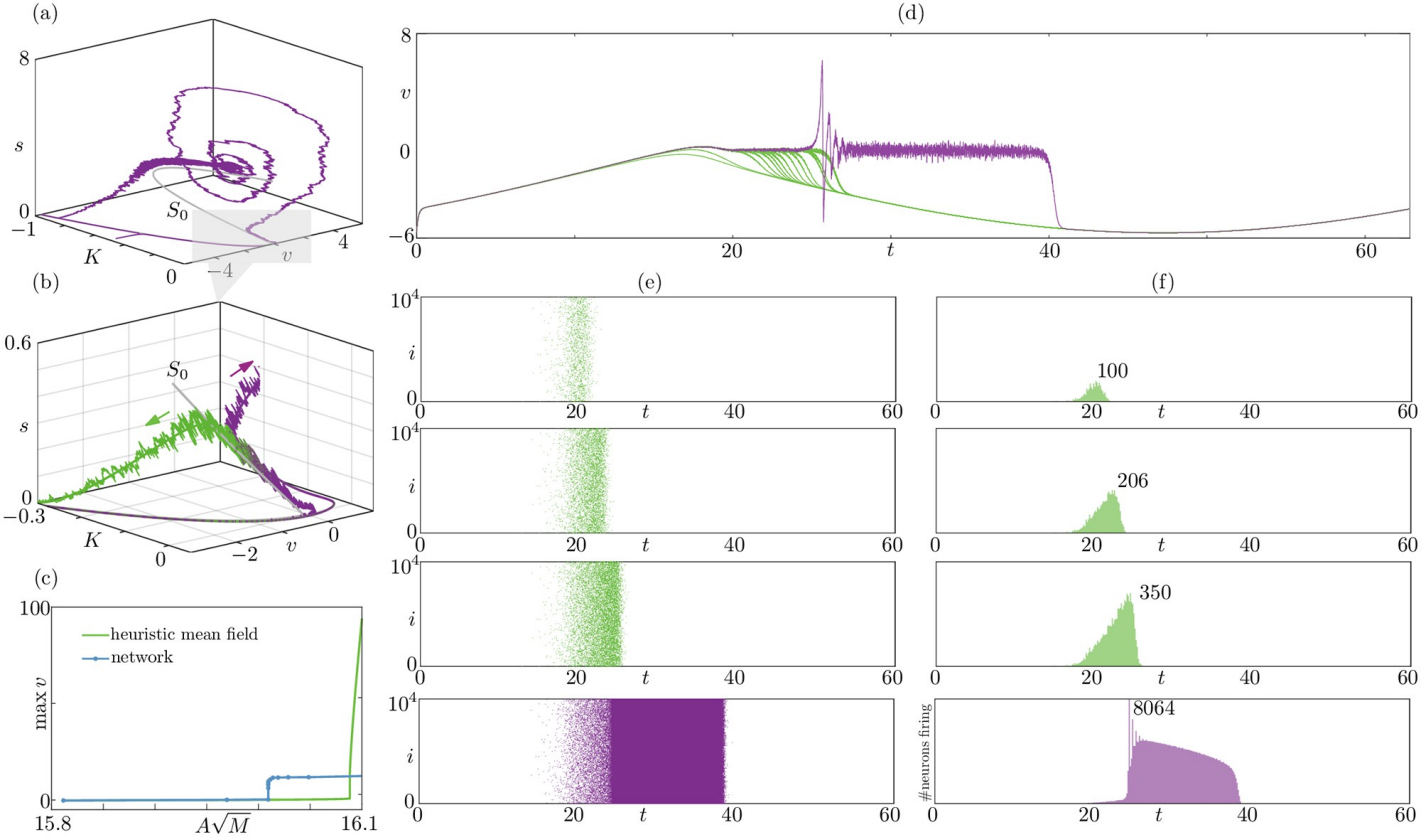
Dynamics of the sparse network (16) with randomly-distributed currents and randomly-distributed connectivity. Panel (a) shows a network bursting solution plotted in the 3D phase space (*K*, *v*, *r*) together with the critical manifold *S*_0_ of the heuristic mean-field system (17). A down-up canard segment is visible in the greyed out region. (b): A zoomed view of panel (a); in addition to *S*_0_ and the bursting solution of panel (a) in purple ($A=16.009453593274596/\sqrt{M}$), we show an orbit with down-down canard segment obtained for slightly perturbed values of *A*, in green ($A=16.009453593274599\sqrt{M}$); we also superimpose solutions of the heuristic mean-field system, whose curves do not have fluctuations, unlike the network ones. Panels (a,b) constitute numerical evidence that a down-to-up canard-mediated transition to bursting exists in this network, as further shown in panels (c–f). (c): Down-to-up route to bursting in the network (blue) and heuristic mean field (green); besides some discrepancies discussed in the main text, both branches contain a quasi-vertical segment typical of canard dynamics, bridging between the non-bursting regime and the bursting one. (d) Representative network solutions along the vertical branch in panel (d), between $A=15.8113883008419/\sqrt{M}$ and $A=16.0094535932746/\sqrt{M}$, displayed in the time series for the voltage *v*; this solutions reveal a clear down-to-up transition, from non bursting (green) to bursting (purple) orbits possessing canard segments (near *v* = 0); the peculiarity of these sparse-network canard solutions is that the associated rate increases rapidly in the canard regime while the mean voltage remains approximately constant, as evidenced in panels (e,f). (e): raster diagram of selected down-to-down (green) and down-to-up (purple) network solutions from (d); the canard segments manifests themselves in the raster plots: their onset coincides with the onset of spiking, and their termination with the jump to the quiescent phase (green) or the start of the tonic phase (purple); along the segment, the network builds up rate (as shown in (f)). (f): histograms of firing events between *t* and *t* + Δ*t*, with Δ*t* = 0.15; along the canard segments the solution increases the firing rate (the longer the segment, the higher the maximum rate in the green histograms); the purple diagram is at a different scale with respect to the green ones, and it represents a bursting solution. Parameters: *N* = 10^4^, *M* = 10^3^, *J* = 1, *τ*_*s*_ = 0.015, Δ_*γ*_ = 0.3, *ε* = 0.1, $\bar{\eta }=-0.5$, Δ = 10^−4^, *v*_*t*_ = −*v*_*r*_ = 100.

A bursting orbit for a network with *N* = 10^4^ neurons is visible in Fig 7a, in the (*v*, *K*, *r*)-space. This simulation is done for a network with sharply peaked current distribution (Δ ≪ 1), which generates a particular bursting pattern, as we will now discuss.

Assuming that the heuristic mean-field description approximates the network simulation, one can reason as in a standard MPR network: the burst is due to a family of foci on the upper branch of an cubic-like critical manifold (visible in grey in the figure). The figure shows that the manifold *S*_0_ of the candidate mean field captures well the geometry of the bursting orbit, and in particular it displays a canard segment along the repelling branch of *S*_0_.

A further inspection of *S*_0_ reveals that, because Δ is small, both folds *F*^±^ of *S*_0_ occur at vanishingly small values of *r*. We observe down-to-down and down-to-up orbits in the network as well as in the heuristic mean field (see Fig 7b), and this suggests the possibility of a continuous down-to-up route to bursting.

It is important to note that the presence of nearby down-down and down-up solutions, on its own, suffices to get a hint of the presence of canards; in this case, we also have an approximating heuristic mean-field description with a computable manifold *S*_0_, which clearly helps us finding values of parameters where the nearby down-down and down-up solution exist; the considerations that follow, however, hold for the finite-size network, even if we obliterate *S*_0_ from Fig 7a and 7b, and from the discussion above.

To uncover a continuous route to bursting *in the network*, we compute several orbits of the system when *A* varies between $A=15.8113883008419/\sqrt{M}$ and $A=16.0094535932746/\sqrt{M}$; while *A* changes, we keep the connectivity matrix *W* constant, that is, we extract it once and reuse it thereafter. The results are given in Fig 7c–7f. In Fig 7d we show the voltage profiles. As anticipated, the canard structure of this network is peculiar, because the currents are almost homogeneously distributed: the canard segment in these orbits stretches along *v* = 0; during this transition, however, the rate increases sharply, as seen in the raster plot Fig 7e and in the histograms in Fig 7f. This means that the excitability threshold for this network occurs for states at constant voltage and progressively large rate, unlike in the cases presented before. This is induced by the fact that the critical manifold *S*_0_ is different between system (17) and system (4). The former is a perturbation of the latter by a term proportional to *s*, which gives rise to a very sharp fold followed by a middle branch along which *v* remains almost constant. This effect is visible on Fig 7a.

In Fig 7c we compare the routes to bursting for the network and the candidate mean field. We find a good agreement in the subthreshold regime, as well as the presence of canard transitions in both systems, marked by quasi-vertical branch segments.

We also notice two types of discrepancies. Firstly, the value of *A* at the quasi-vertical segment differs slightly in the *N* = 10^4^ and in the mean field; this is to be expected, given that the mean field is only heuristic. Solution types along the canard explosion, however, are very similar in the two systems. Secondly, we notice a discrepancy in the maximum voltage *v*, which is the solution measure chosen for the network, especially in the bursting regime. This type of discrepancy is not unexpected: in the finite-size network, each neuron has a voltage at most equal to the reset value, hence the maximum mean voltage is capped in the network; the heuristic mean field, on the other hand, supports solutions with a very large maximum voltage.

As anticipated, the discrepancy in Fig 7c is relevant if one wants to assess the accuracy of the mean field in the bursting regime, but is immaterial for the canard-mediated route to bursting: the blue route in Fig 7c, and the data in Fig 7d–7f show such route independently of the existence of *S*_0_, or of the accuracy of the heuristic mean field.

## 5 Conclusions

The geometry of excitability and transition to bursting behavior in single neurons and allied systems is governed by canard solutions, which act as thresholds and determine the response of the system to slow parametric changes. We have shown that this structure carries over in the mean-field limit of large populations of excitable cells, as well as in large finite systems.

In these cases, the average voltage of the population plays the same role as the voltage in the single cell, and a well defined rate emerges as a new macroscopic variable. If a separation of time scales exists between external input and voltage at the level of a single cell, such separation persists at network level, between the input and the coupled mean voltage and rate.

Two main results have been discovered at network level: (i) a large class of networks of QIF neurons subject to an external stimulus with Cauchy-distributed background currents undergoes a continuous transition to bursting (either up-to-down, or down-to-up) upon increasing the forcing amplitude; to the best of our knowledge, this statement holds for any QIF network amenable to the Ott-Antonsen reduction currently derived in the literature, that is, expressible as system (9). (ii) The canard-mediated route to bursting is present also in sparse networks, for which there is no exact limit; obtaining an approximate mean-field limit is convenient to compare network trajectories to low-dimensional manifolds, but it is not strictly necessary, and in fact continuous routes to bursting are present in small networks too.

Since QIF neurons are general representatives of type-I neurons, we expect that similar properties will survive in networks of more realistic cells, up to their mean field-limit, which need not be an ODE. Introduction of inhibitory networks could provide a connection between this work and the concept of balanced networks where, depending on the details of the connection strengths, the firing is either driven by the mean input (analogous to our tonic behavior) or by the fluctuations (analogous to the excitable case). Another open interesting research direction is to investigate heterogeneous networks whose distributions are asymmetric, thereby violating the assumption required for the Ott-Antonsen exact mean-field derivation: without a mean field, we could still be able to observe a contraction of the dynamics to a low-dimensional manifold, and a canard-mediated excitability threshold. There is currently no available theory for such cases, except for continuous coarse-grained networks [36, 40], but a numerical exploration of the averaged voltages and synaptic variables may reveal an underlying low-dimensional structure, similarly to what has been found in spatially-extended neural field models.
